# Supplementary material for: Chromatin regulates IL-33 release and extracellular cytokine activity
Source: Nat Commun. 2018 Aug 14;9:3244. doi: 10.1038/s41467-018-05485-x (PMC6092330; doi:10.1038/s41467-018-05485-x)
Supplement: Supplementary file 1 — Supplementary Information [file 41467_2018_5485_MOESM1_ESM.pdf]

SUPPLEMENTARY INFORMATION

Chromatin regulates IL-33 release and bioactivity

Travers et al

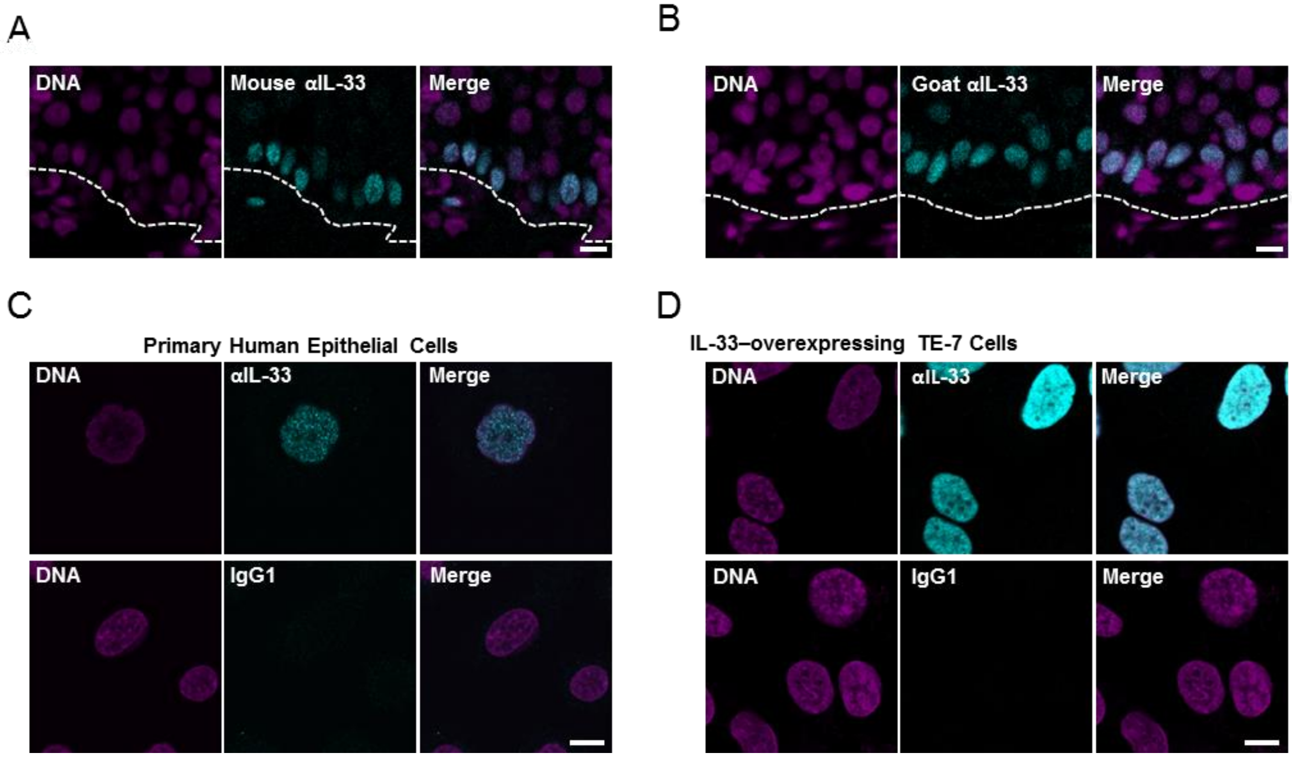

Supplementary Figure 1: Nuclear localization of IL-33 in esophageal epithelial cells. (A) (A,B) Immunofluorescence of esophageal biopsies patients with active EoE. **(C,D)** Immunofluorescence of primary human esophageal epithelial cells (C) or TE-7 cells with stable, constitutive overexpression of wild-type IL-33 (D). For (A-D), left column is DNA as indicated by DAPI staining (magenta); the middle column is staining (turquoise) with mouse anti–IL-33 antibody (A,C,D), goat anti–IL-33 antibody (B), or antibody controls (A-D) as indicated; and the right column shows the merged images. Scale bar is 10 µm. DAPI, 4',6-diamidino-2-phenylindole; EoE, eosinophilic esophagitis; IL, interleukin.

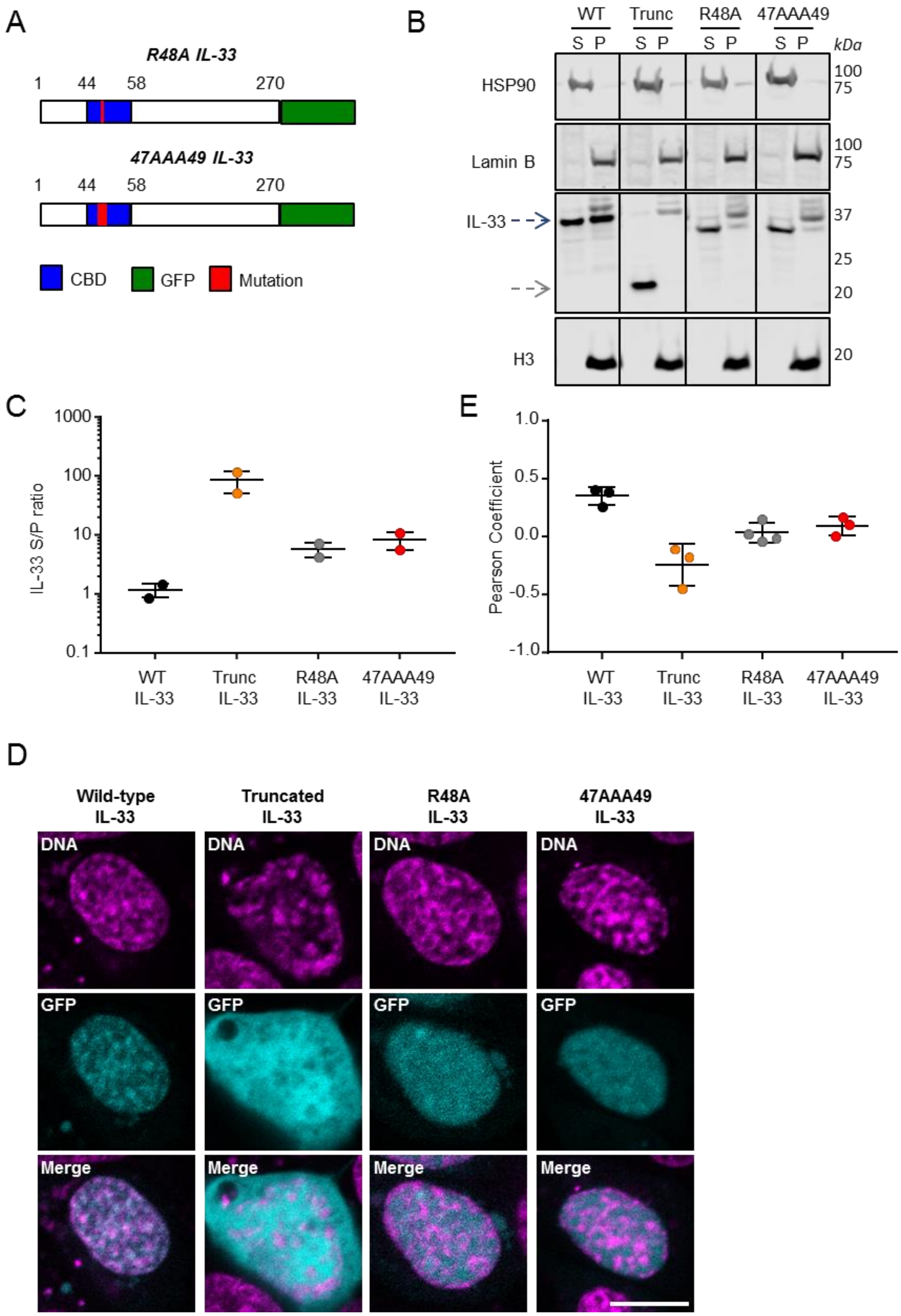

Supplementary Figure 2: IL-33 mutants exhibit decreased chromatin binding. (A) Schematic of 47AAA49 and R48A IL-33-GFP fusion proteins. **(B,C)** 293T cells were transiently

transfected with plasmid encoding WT IL-33 or IL-33 structural variant as indicated and were treated with Triton X-100 (0.5%). (B) is a representative Western blot, and (C) shows quantification of the ratio of IL-33 detected in the supernatant to pellet (S/P) after Triton X-100 treatment from two independent experiments. Depicted is mean ± standard error of the mean. Blue arrow indicates expected size of the full-length IL-33 protein, and gray arrow indicates expected size of the truncated IL-33 protein. **(D)** Representative live-cell images from one experiment from (E). Top row indicates Hoechst 33342 DNA dye (magenta), middle row indicates presence of GFP-fusion protein (turquoise), and bottom row contains merged images. Scale bar is 10 μm. **(E)** Quantification of the Pearson correlation coefficient within the nucleus between fluorescence from Hoechst 33342 DNA dye and GFP after transient transfection of plasmid encoding indicated GFP-fusion protein into TE-7 cells. Graph shows the average and standard deviation of combined data from two independent experiments. GFP, green fluorescent protein; IL, interleukin; Trunc, truncated.

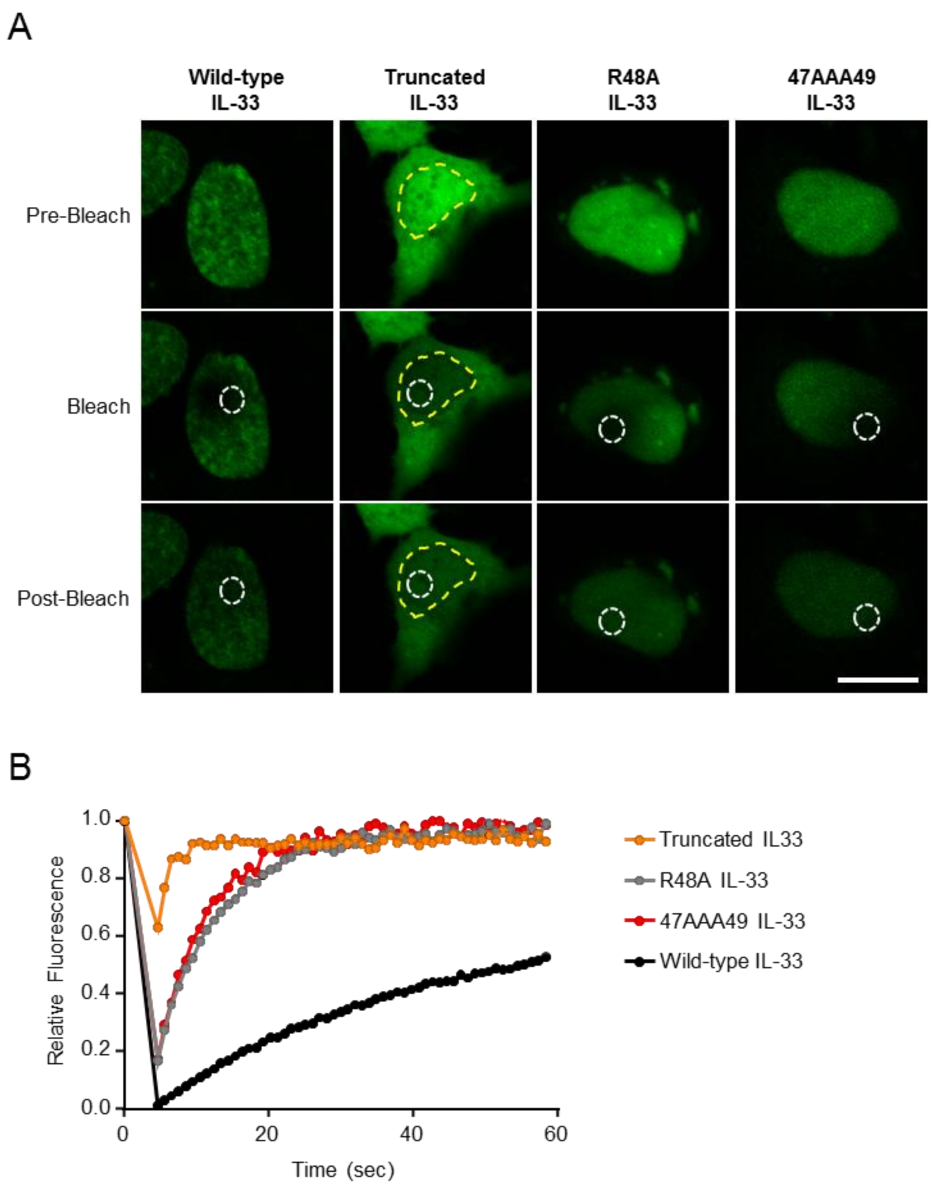

Supplementary Figure 3: Dynamics of chromatin binding of IL-33 mutants. (A) Fluorescence recovery after photobleaching (FRAP) was performed on TE-7 cells after transient transfection with plasmid encoding the indicated GFP-fusion proteins. A region of interest (ROI) [white dashed lines] was bleached, and fluorescence within that ROI was determined continuously over the following 60 seconds. Representative images of GFP fluorescence (green) from before bleach (top row), immediately after bleach (middle row), and 60 seconds post-bleach (bottom row) are shown for the indicated GFP-fusion protein. For GFP-fusion proteins with significant cytoplasmic localization, the nucleus is outlined with yellow dashed lines. Scale bar is 10 µm. **(B)** Representative FRAP experiment showing fluorescence within ROI during bleach and for 60 seconds post-bleach for each GFP-fusion protein. Please note that 70% recovery times determined in this experiment cannot be directly compared with those determined in FRAP experiments in Figure 4 because increased depth of bleach which resulted from use of a more powerful laser. GFP, green fluorescent protein; IL, interleukin.

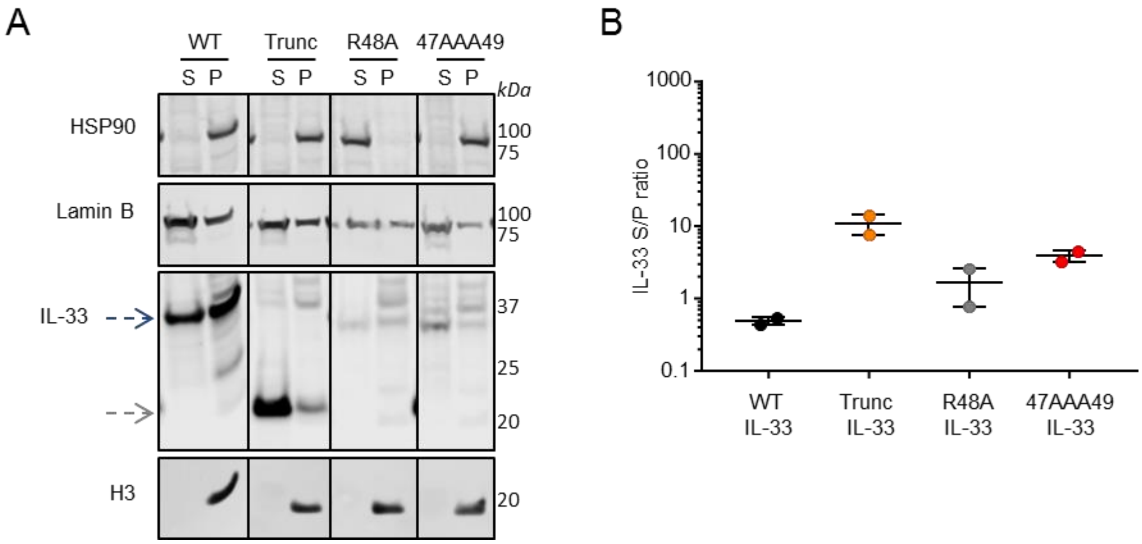

Supplementary Figure 4: IL-33 mutants exhibit increased release during necrosis. (A,B) 293T cells were transiently transfected with plasmid encoding indicated GFP-fusion protein and then subjected to cryoshock. A demonstrates a representative Western blot, and B demonstrates the ratio of indicated GFP-fusion protein detected in supernatant to pellet. Mean ± standard error of the mean of two independent experiments are depicted. Blue arrow indicates expected size of the full-length IL-33 protein, and gray arrow indicates expected size of the truncated IL-33 protein. GFP, green fluorescent protein; IL, interleukin.

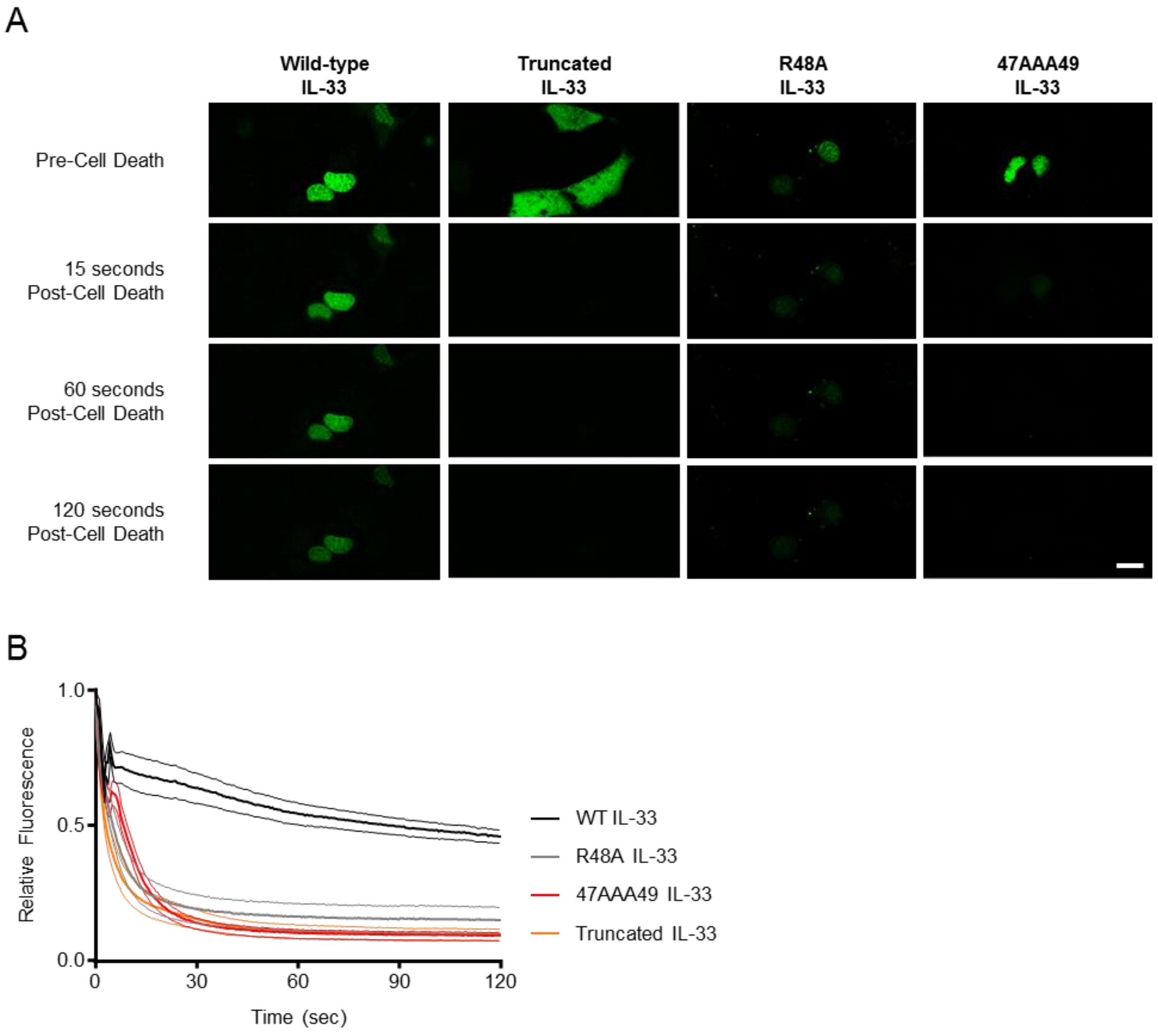

Supplementary Figure 5: IL-33 mutants exhibit more rapid release during necrosis. (A) TE-7 cells were transiently transfected with plasmid encoding the indicated GFP-fusion protein and then treated with Triton X-100 (0.13%) to induce necrosis. Depicted are images from a representative experiment showing 5 seconds prior to cell death by the Triton X-100 ("Pre-Cell Death"; top row) and 15, 60, and 120 seconds after death ("Post-Cell Death"; second, third, and fourth rows, respectively). Scale bar is 10 µm. **(B)** Quantification of intracellular fluorescence intensity from two combined independent experiments. Data are normalized to time zero, which is when Triton X-100 was added. For each depicted curve, the central, thicker line indicates the mean and the bordering, thinner lines of the same color indicate the standard error of the mean. GFP, green fluorescent protein; IL, interleukin.

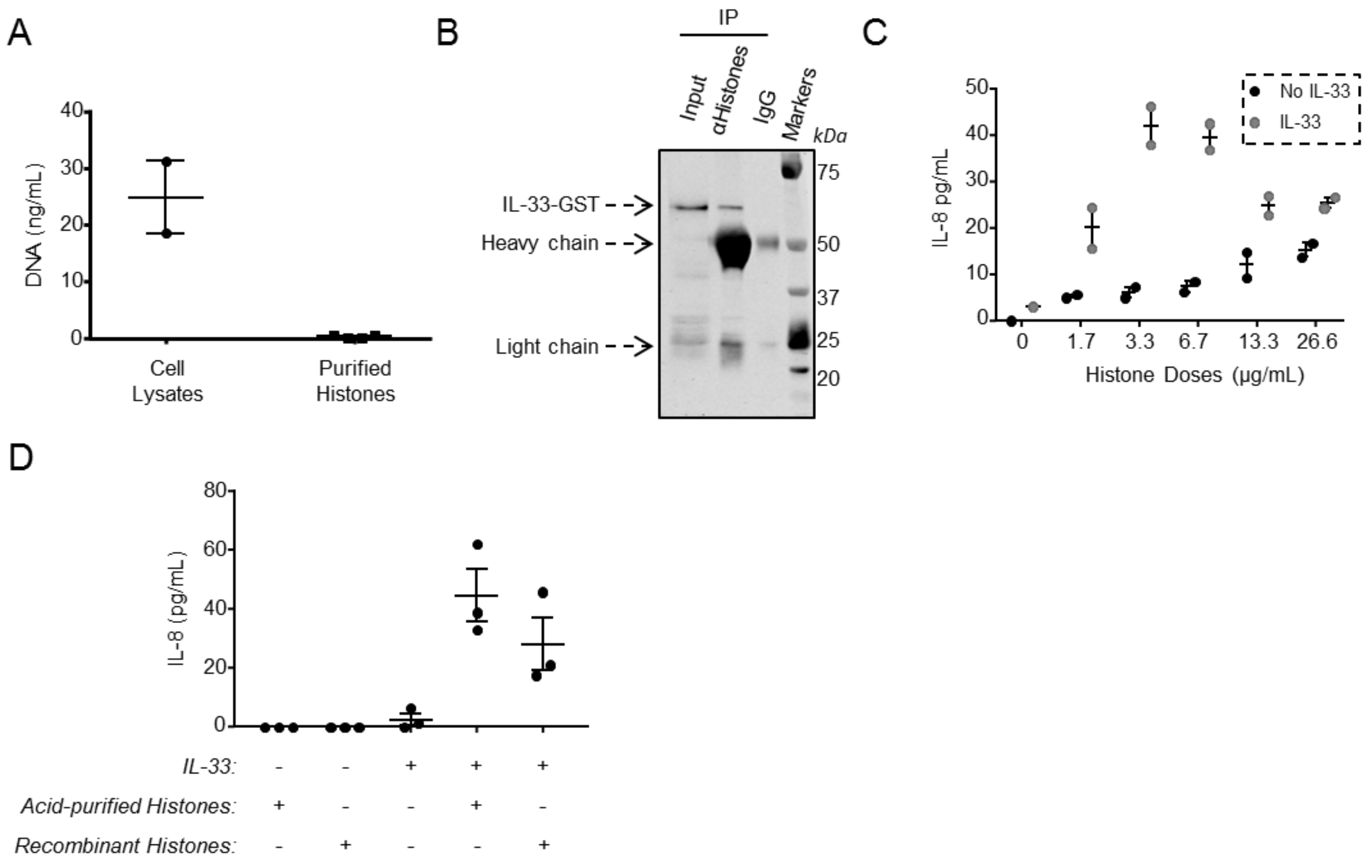

Supplementary Figure 6: Further characterization of acid-purified histones. (A) DNA concentration in TE-7 cell lysates and acid-purified histones as determined by Qubit. **(B)** Acid-extracted histones and recombinant WT IL-33-GST were incubated together in the presence of DNAse and then co-immunoprecipitated with anti-histone H2B and anti-histone H2A antibodies (αhistones) or isotype controls (IgG). Expression of GST was then assessed by Western blot analysis. **(C)** IL-8 levels in supernatants of HMC-1 mast cells treated overnight with 11.7 nM recombinant WT IL-33 or vehicle and indicated amounts (in μg/mL) of acid-purified histones. **(D)** IL-8 levels in supernants of HMC-1 mast cells treated overnight with 11.7 nM recombinant WT IL-33, 6.7 μg/mL purified acid-extracted histones, or 6.7 μg/mL recombinant histones as indicated. C and D depict mean ± standard error of the mean of data that is either representative of two experiments (C) or combined from two independent experiments. GST, glutathione S-transferase; Ig, immunoglobulin; IL-33, interleukin; ST2, suppressor of tumorigenicity 2; WT, wild-type.

Figure 1:

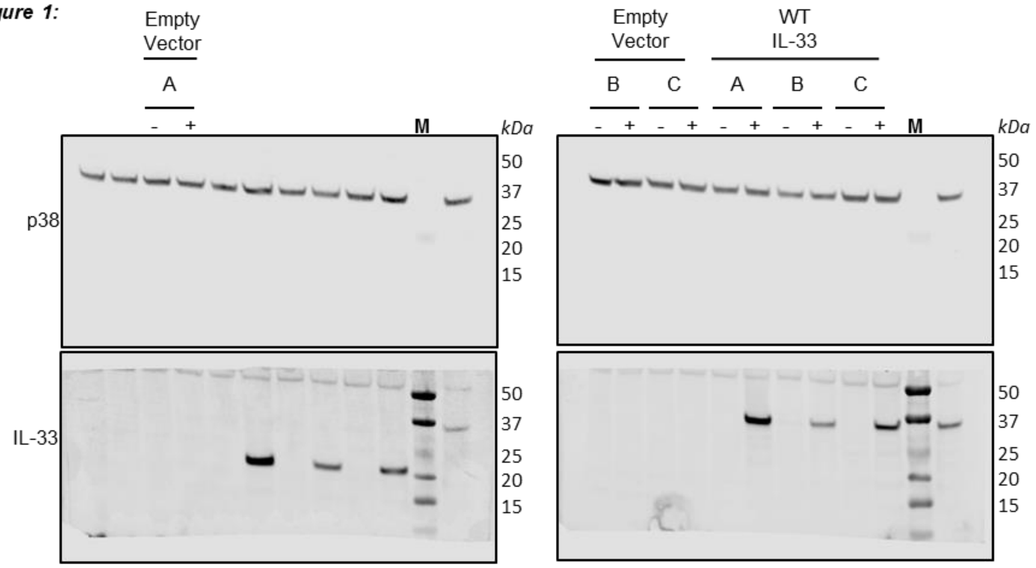

Figure 2:

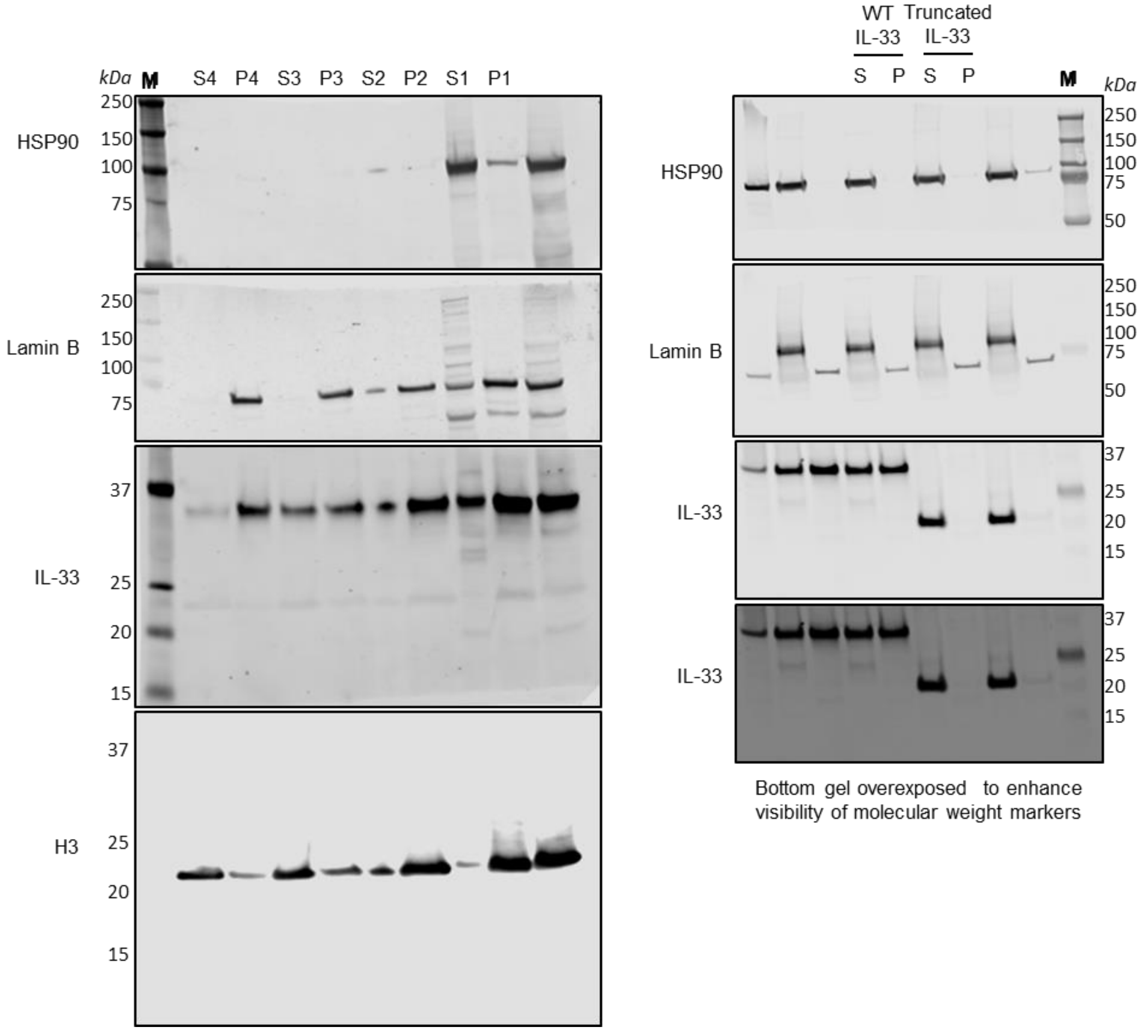

Bottom gel overexposed to enhance visibility of molecular weight markers

Supplementary Figure 7: Uncropped Western blots from Figures 1 and 2. Bold M indicates lane with containing molecular weight markers. Unlabeled lanes contain irrelevant samples. Please see legends for Figures 1 and 2 for more information regarding the samples present in each lane.

Figure 5:

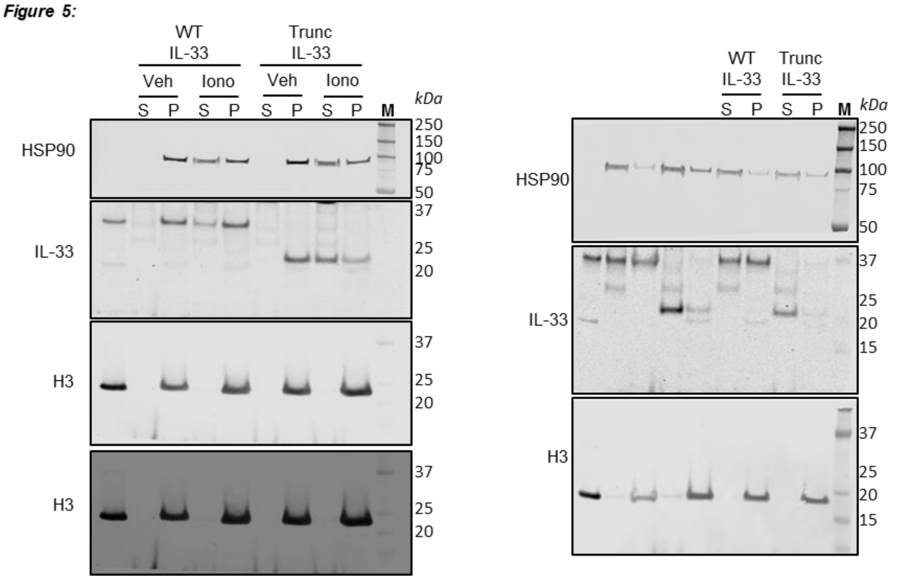

Bottom gel overexposed to enhance visibility of molecular weight markers

Figure 7:

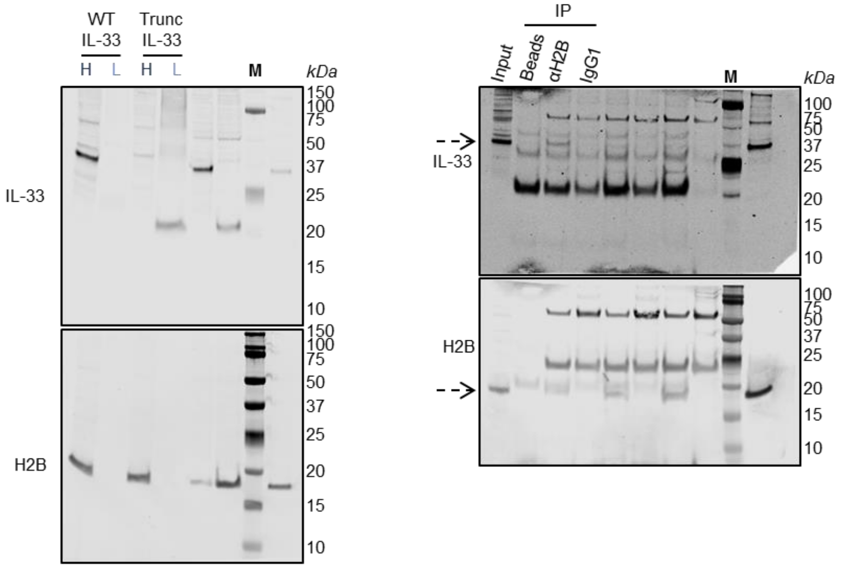

Figure 8:

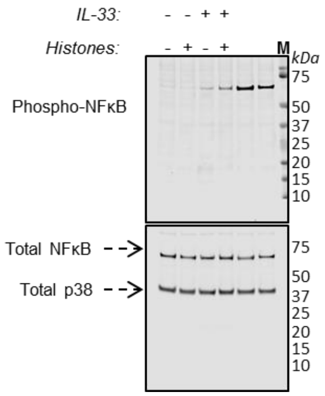

Supplementary Figure 8: Uncropped Western blots from Figures 5, 7 and 8. Bold M indicates lane with containing molecular weight markers. Unlabeled lanes contain irrelevant samples. Please see legends for Figures 5, 7 and 8 for more information regarding the samples present in each lane.

Supplementary Figure 2:

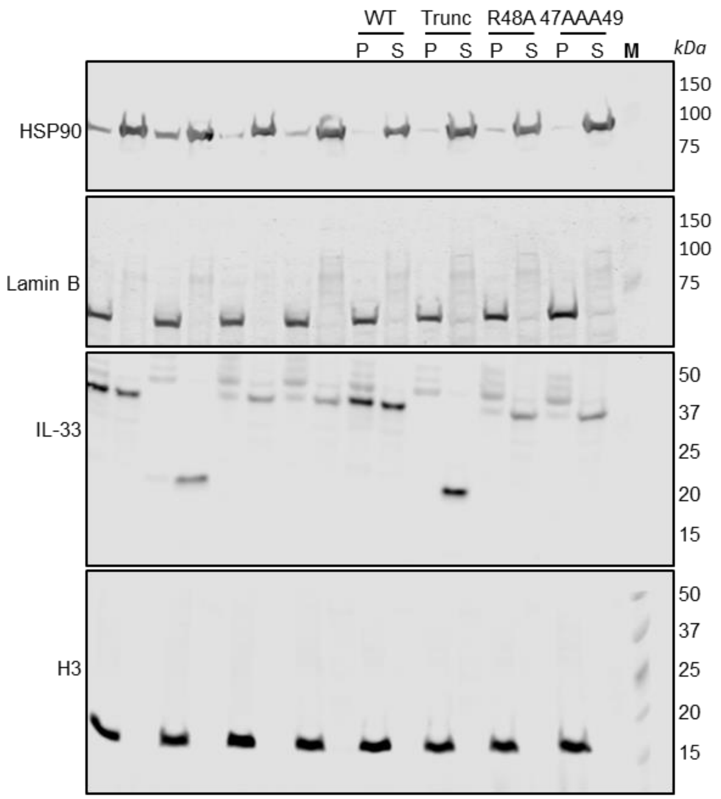

Supplementary Figure 4:

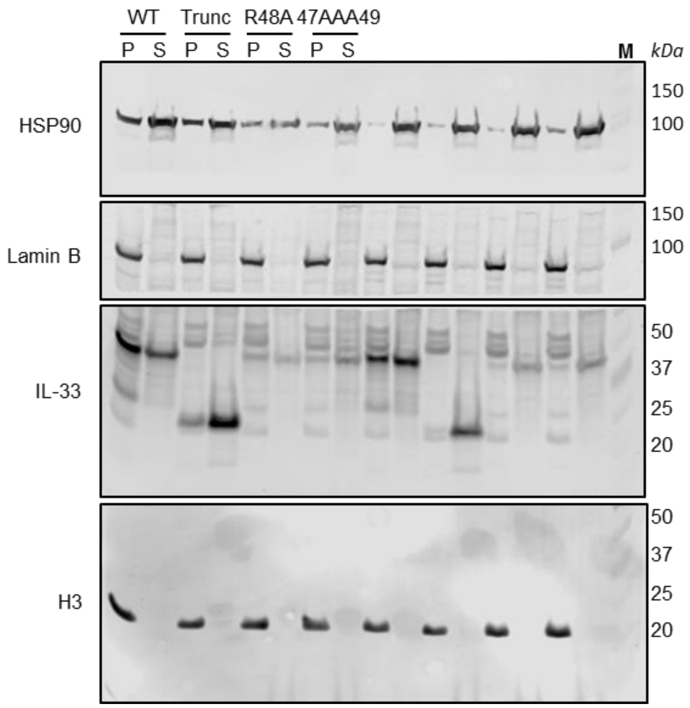

Supplementary Figure 9: Uncropped Western blots from Supplementary Figures 2 and 4. Bold M indicates lane with containing molecular weight markers. Unlabeled lanes contain irrelevant samples. Please see legends for Supplementary Figures 2 and 4 for more information regarding the samples present in each lane.

	**# DEG**	**Names**
Wild-type IL-33	1	*IL33*
Empty Vector	4	*MMP13, DOCK1, UBE2F-SCLY, TMX2-CTNND1*

Supplementary Table 1: Differentially expressed genes after overexpression of IL-33. Table of genes differentially expressed (DEG) in all three clones overexpressing WT IL-33 or the empty vector, respectively, from experiment in Figure 1. Criteria were RPKM ≥ 1 in at least one sample, FDR $p < 0.05$, and fold change ≥ 1.5. FDR, false discovery rate; IL, interleukin; RPKM, reads per kilobase of pranscript per million mapped reads; WT, wild-type.

**Protein**	**50% Recovery**	**70% Recovery**
GFP	N.D. (I.B.)	N.D. (I.B.)
Truncated IL-33	N.D. (I.B.)	N.D. (I.B.)
IL-1α	N.D. (I.B.)	3.5 ± 0.2 sec
Wild-type IL-33	13.9 ± 1.0 sec	29.7 ± 2.3 sec
Histone H2B	N.D. (I.R.)	N.D. (I.R.)

Supplementary Table 2: Quantitation of intranuclear mobilities. Mean and standard error of the mean of 50% and 70% recoveries of indicated GFP-fusion proteins from 3-5 independent experiments of fluorescence recovery after photobleaching (FRAP) from Figure 4. GFP, green fluorescent protein; IL,interleukin; N.D. (I.B.), not determined due to insufficient bleach; N.D. (I.R.), not determined due to insufficient recovery; sec, second.

**Protein**	**70% Recovery**
Truncated IL-33	N.D. (I.B.)
R48A IL-33	14.7 $\pm$ 1.3 sec
47AAA49 IL-33	14.1 $\pm$ 1.3 sec
WT IL-33	N.D. (I.R.)

Supplementary Table 3: Quantitation of intranuclear mobilities of IL-33 mutants. (C) Mean and standard error of the mean of 70% recoveries of indicated GFP-fusion proteins from 2 independent experiments from Supplementary Figure 3. N.D. (I.B.), not determined due to insufficient bleach; N.D. (I.R.), not determined due to insufficient recovery; WT, wild-type.

**Protein**	**15 sec**	**60 sec**	**120 sec**
Truncated IL-33	21.3 ± 4.4%	10.8 ± 2.4%	9.6 ± 2.1%
R48A IL-33	25.3 ± 5.4%	16.4 ± 4.8%	14.9 ± 4.7%
47AAA49 IL-33	27.3 ± 3.5%	10.1 ± 1.5%	9.1 ± 1.4%
WT IL-33	68.1 ± 6.3%	54.3 ± 3.9%	45.8 ± 2.5%

Supplementary Table 4: Quantitation of kinetics of release of IL-33 mutants. Mean and standard error of the mean of the percentage of intracellular fluorescence intensity at indicated timepoints from Supplementary Figure 5 relative to time zero. IL, interleukin; sec, second.

	Untreated		Histones		IL-33		IL-33 + Histones	
Cytokine	1	2	1	2	1	2	1	2
**MCP-1/CCL2**	248.39	299.15	236.85	272.96	3345.8	1275.86	224925.49	225416.60
**CCL4**	1.18	3.63	1.71	3.63	169.64	78.77	631.86	532.37
**CCL3**	9.09	5.39	6.62	7.38	108.55	52.22	438.25	357.39
**IL-8**	5.16	0.99	1.34	5.34	81.68	24.45	330.77	183.86
**IL-3**	83.33	56.4	77.52	57.81	179.98	67.13	288.12	88.82
**GRO α**	51.81	21.66	27.05	39.33	34.73	19.5	54.18	67.84
**CCL5**	21.43	15.48	12.28	24.52	22.12	13.4	36.58	33.24
**FGF-2**	41.02	24.96	8.8	34.85	21.06	16.19	0.1	29.34
**IL-6**	0.1	0.1	0.1	0.23	6.63	3.23	52.5	26.05
**IL-1RA**	267.97	295.25	19.06	246.21	27.4	10.89	30.94	14.94
**CCL22**	49.78	20.93	8.61	18.47	23.39	35.58	0.1	11.07
**PDGF-AA**	4.97	3.82	3.8	5.17	9.92	5.93	16.61	10.43
**IP-10**	2.45	0.1	0.1	0.1	21.36	3.21	32.23	9.6
**GM-CSF**	1.43	0.1	0.1	0.54	1.52	0.64	5.59	5.39
**IL-12P40**	1.96	3.95	1.96	3.2	2.95	3.45	4.94	4.94
**EGF**	0.1	0.9	2.66	5.23	2.66	0.1	8.2	4.36
**Flt-3L**	3.82	0.16	2.24	3.07	2.53	2.24	3.82	3.07
**MCP-3**	2.4	2.03	1.33	2.4	3.64	0.3	1.33	2.03
**TGF-a**	4.3	2.14	2.69	2.22	3.5	1.62	3.75	2.02
**IL-18**	10.67	8.27	2.31	11.27	3.5	1.56	2.31	2.01
**PDGF-BB**	3.87	1.2	1.06	3.87	0.63	1.76	0.1	1.76
**IFNα2**	2.82	2.82	2.3	3.33	1.28	2.3	0.51	1.28
**IL-12P70**	1.03	0.88	0.35	0.59	1.27	0.88	0.98	1.08
**IL-15**	0.55	0.9	0.64	0.9	0.64	0.21	1.07	0.55
**CX3CL1**	25.24	7.4	3.2	10.79	0.69	0	5.71	0
**Eotaxin-1**	OOR <	OOR <	OOR <	OOR <	OOR <	0.64	OOR <	OOR <
**sCD40L**	OOR <	OOR <	OOR <	OOR <	OOR <	OOR <	OOR <	OOR <
**IL-17A**	OOR <	OOR <	OOR <	OOR <	OOR <	OOR <	OOR <	OOR <
**G-CSF**	0.15	OOR	OOR	OOR	OOR	OOR	OOR	OOR
**IFNγ**	OOR	OOR	OOR	OOR	OOR	OOR	OOR	OOR
**IL-10**	0.1	OOR	0.1	0.1	OOR	OOR	0.37	OOR
**IL-1a**	OOR	OOR	OOR	OOR	OOR	OOR	OOR	OOR
**VEGF-A**	OOR <	OOR <	OOR <	OOR <	4.86	2.07	7.09	1.1
**IL-1B**	0.32	0.15	0.4	0.13	0.61	0.42	0.96	0.69
**IL-9**	0.3	0.18	0.29	0.33	0.3	0.28	0.34	0.35
**TNFa**	0.03	0.04	0.15	0.08	0.31	0.09	0.64	0.23
**IL-4**	2.65	0.4	0.15	1.13	0.64	0.15	2.13	0.15

**IL-13**	0.13	OOR <	OOR <	OOR <	OOR <	0.01	0.13	0.13
**TNFB**	0.49	0.2	0.4	0.35	0.54	0.1	0.35	0.1
**IL-7**	0.72	0.09	OOR <	0.81	0.22	0.15	0.39	0.09
**IL-5**	0.05	0.02	0.01	0.03	0.03	0.03	0.09	0.05
**IL-2**	0.26	0.1	0.12	0.08	0.44	0.41	0.18	0.04

Supplementary Table 5: Concentration of individual cytokines. Table depicts expression (in pg/mL) of indicated proteins in supernatants of cells given indicated treatments in samples from Multiplex in Figure 8B. Grey text indicates cytokines not significantly affected by IL-33 and histones. OOR, out of range; OOR>, out of range above the 4 or 5 parameter logistic standard curve; OOR<, out of range below the 4 or 5 parameter logistic standard curve; IL, interleukin.

Cytokine	Relative to Untreated				(IL-33+Histones)/(IL-33)
	Untreated	Histones	IL-33	IL-33 +Histones	
MCP-1/CCL2	1.0	0.9	8.4	822.5	97.9
Fractalkine/CX3CL1	1.0	0.4	0.02	0.2	10.0
IL-6	1.0	1.7	49.3	392.8	8.0
GM-CSF	1.0	0.4	1.4	7.2	5.1
MIP-1α/CCL3	1.0	1.0	11.1	54.9	4.9
IL-8	1.0	1.1	17.3	83.7	4.8
MIP-1Β/CCL4	1.0	1.1	51.6	242.0	4.7
EGF	1.0	7.9	2.8	12.6	4.5
GRO alpha	1.0	0.9	0.7	1.7	2.4
RANTES	1.0	1.0	1.0	1.9	1.9
IL-15	1.0	1.1	0.6	1.1	1.8
PDGF-AA	1.0	1.0	1.8	3.1	1.7
IP-10	1.0	0.1	9.6	16.4	1.7
IL-12P40	1.0	0.9	1.1	1.7	1.5
IL-3	1.0	1.0	1.8	2.7	1.5
Flt-3L	1.0	1.3	1.2	1.7	1.4
TGF-a	1.0	0.8	0.8	0.9	1.1
IL-12P70	1.0	0.5	1.1	1.1	1.0
IL-1RA	1.0	0.5	0.1	0.1	1.0
MCP-3	1.0	0.8	0.9	0.8	0.9
PDGF-BB	1.0	1.0	0.5	0.4	0.8
FGF-2	1.0	0.7	0.6	0.4	0.7
IL-18	1.0	0.7	0.3	0.2	0.7
IFNa2	1.0	1.0	0.6	0.3	0.5
MDC	1.0	0.4	0.8	0.2	0.3

Supplementary Table 6: Normalized expression of individual cytokines. Table indicates fold-change of expression of indicated proteins in supernatants of cells given indicated treatments as compared to untreated cells in samples from Multiplex in Figure 8B. IL, interleukin.
